# From genes to networks: neurobiological bases of neurodiversity across common developmental disorders

**DOI:** 10.3389/fnins.2026.1761279

**Published:** 2026-02-19

**Authors:** Shiqi Ding

**Affiliations:** NUS High School of Math and Science, Singapore, Singapore

**Keywords:** brain circuit maturation, genetic architecture, neurodevelopmental disorders, neuroplasticity, synaptic signaling pathways

## Abstract

Neurodiversity is a category for many NDDs, ranging from ASD to OCD. The Clinical Symptoms associated with NDDs may differ, but have many common features. Hence, there are many biological similarities between the various Neurodevelopmental Disorders. It has been assumed that one cause of these commonalities is due to shared Etiologies. This review provides information about Genes (Genetics), Proteins (Molecular), and Biology. Together, they provide evidence for common mechanisms (convergent) and Different mechanisms (divergent) across all Neurodevelopmental Disorders. These Types often share Polygenic Genes with Rare and Common Variants. The Shared Genes are commonly involved in the regulation of Synaptic Function, Chromatin Remodeling, and Immune Pathways. In addition, NDDs often have disrupted pathways associated with mTOR, Wnt/Beta-Catenin, and Immune Pathways. We also consider the Impact of Spatial and Temporal Gene Expression on NDD development across different stages (from Prenatal Development to Postnatal Development). We also discuss the impact of disruption during Prenatal and Postnatal Maturation of the Brain, including alterations in the processes of Neurogenesis, Synaptic Pruning, and Neural Circuit Integration. Based on these findings, we present a systems-based, multi-level approach to help understand the biological basis of neurodiversity, along with future research directions on Therapeutic Strategies across Diagnostic Groups, Developmentally Informed Interventions, and the Development of Inclusive Clinical and Societal Practices.

## 1 Introduction

Although the term neurodiversity implies a neurological origin, it was initially developed by sociologist Judy Singer in the late 1990s, conceptualizing neurological conditions as natural variations in human behavior, cognition, and perception rather than inherently pathological states (Chapman, 2021). The neurodiversity movement advocates for the acceptance and inclusion of atypically developing individuals by reframing conditions like autism spectrum disorder (ASD), attention-deficit/hyperactivity disorder (ADHD), dyslexia, and obsessive-compulsive disorder (OCD) to highlight their place within the normal spectrum of human variation (Singer, 1999).

### 1.1 Conceptual background and transdiagnostic perspective

This review focuses on ASD, ADHD, dyslexia, and OCD due to their high prevalence and shared neurobiological pathways, making them key representatives of neurodevelopmental disorders (NDDs). ADHD is characterized by sustained patterns of inattention, hyperactivity, and impulsivity, leading to challenges in maintaining attention and managing behavior. ASD is defined by deficits in social interaction and communication, along with restricted and repetitive behaviors. Both disorders often exhibit comorbidity and share similar overall neurological and genetic pathways (Martinez et al., 2024; Hosseini and Molla, 2025; Snowling, 2001).

Dyslexia, often regarded as a learning difficulty, results from challenges with phonological processing, rather than intelligence or hearing deficits (Carlisi et al., 2017). Moreover, OCD, while classified as a psychiatric disorder, overlaps socially and neurologically with ASD and ADHD, particularly in cortico-striato-thalamo-cortical neural circuits (Musiek et al., 2018). Additionally, a significant number of individuals with these conditions present with central auditory processing disorder (CAPD), further complicating diagnosis and treatment (Gerdts and Bernier, 2011).

Epidemiological studies reveal substantial co-morbidity rates: approximately 50–70% of individuals with ASD are also diagnosed with ADHD, 25–40% of ADHD children have dyslexia, and one-third of children with ASD concurrently exhibit dyslexia (Carrascosa-Romero and De Cabo De La Vega, 2015; Young et al., 2020). These statistics underscore the need to explore shared etiologies and neurobiological mechanisms that may unify these distinct disorders. Advances in genetics and molecular neuroscience have identified common risk loci, signaling pathways, neurotransmitter systems, and dysregulated brain network connectivity, uniting previously distinct diagnostic categories.

Recognizing the breadth of neurodiversity, this review emphasizes ASD and ADHD for deeper analysis due to their well-documented co-occurrence, shared heritability, and converging biological mechanisms. By investigating genetic, neuroimmune, and synaptic mechanisms, we aim to present an integrated model of genetic variation and its neurological impacts, with practical implications for refining diagnostic criteria and developing targeted interventions.

Genomic studies reveal shared risk loci in individuals with Asperger-like profiles, including broader ASD loci (e.g., 3p14, 7q32) and unique potential variants (e.g., 15q22, NTRK3), indicating the diversity within these conditions. This review discusses biological, developmental, and genetic factors contributing to the co-occurrence of ASD and ADHD as significant neurological categories and concludes with recommendations for therapeutic approaches.

### 1.2 Rationale for focusing on ASD and ADHD

Of the many genetic and environmental influences on development, the two that have received the most attention in recent years are the ASDs and ADHD’s. Studies using large datasets, including Family-Based Genomic Sequencing Studies and Genome-Wide Association Studies (GWAS), have generated a wealth of genetic data. This genetic data has allowed for extensive studies on the heritability, polygenic risk scores, and Pleiotropy (the association of genetic risk with more than one diagnosis) of both ASD and ADHD.

Both ASDs and ADHDs are also at the cutting edge of research in Multi-Omics (i.e., Gene Expression, Transcriptomes, Epigenomes, Brain Imaging). This research has allowed investigators to understand, at a deeper level, the development of synapses, the role of Neuroimmunology, and the Organizing of Brain Circuits across multiple stages of Development. Additionally, ASD and ADHD have some of the highest Co-Morbidity burdens of any of the Neurodevelopmental Disorders, with ASD frequently comorbid with one another and with Learning Disorders and Compulsive Disorders, thus providing a strong empirical basis for Trans-Diagnostic Research.

ASD’s and ADHD’s have great Trans-Diagnostic Value in terms of how they can be used to help model the Diversity of Neurodevelopmental Outcomes. ASD’s and ADHD’s encompass a wide range of different Cognitive, Behavioral and Functional Outcomes but share many of the same Neurobiological Substrates. Thus, studying and developing models of trans-diagnostics using these two conditions allows the development of a framework for linking Genetic Variability with Molecular Pathways and Developmental Timing to Neural Circuitry, with implications that extend beyond the individual diagnosis and encompass the full breadth of the Neurodiverse Spectrum (Mattheisen et al., 2022; Yousaf et al., 2020).

## 2 Genetic architecture of neurodiverse conditions

### 2.1 Polygenic architecture and heritability across neurodiverse conditions

Neurodiverse conditions, such as autism spectrum disorder (ASD), attention-deficit/hyperactivity disorder (ADHD), dyslexia, and obsessive-compulsive disorder (OCD), have high heritability (40–80%), suggesting genetic factors play a central role in their emergence (Eyring and Geschwind, 2021). Each condition presents its own unique cognitive and behavioral profiles, but genetic studies are identifying shared and divergent architectures from both rare and common variants.

One of the defining characteristics of these disorders is polygenicity: common single-nucleotide polymorphisms (SNPs) of small effect can contribute to a large portion of overall risk. In ASD, it is estimated that over 50% of liability is attributable to common variation, yet genome-wide association studies (GWAS) have identified relatively few genome-wide significant loci (Grove et al., 2019; Franke et al., 2009; Demontis et al., 2019). ADHD has also been shown to sit largely on a polygenic structure, and recently, two large GWAS in ADHD identified 27 loci and reported high genetic correlations with other behavioral traits like risk-taking and substance use (Eising et al., 2022). Dyslexia has been studied for years using linkage and candidate-gene approaches (Szejko et al., 2021; Strom et al., 2024), and more recently, GWAS has identified 42 loci that reached significance across cognitive ability and educational attainment measures. While historically there had been fewer genetic studies, OCD has recently shown SNP-based heritability estimates of 6–28%, and a GWAS meta-analysis identified 30 loci associated with risk, many clustered in excitatory cortical neurons (Smit et al., 2020; Demontis et al., 2023; Koller et al., 2024).

### 2.2 Cross-disorder genetic correlations and dimensional risk models

Cross-disorder analyses support significant pleiotropy (meaning something affects multiple traits). ASD and ADHD share many genetic correlations (Gialluisi et al., 2021; Strom et al., 2024), and common variation has been shown to correlate with cognitive measures for dyslexia as well. The polygenic risk score (PRS) for dyslexia was shown to have a negative correlation with ADHD, but a positive correlation with cognitive measures (Eising et al., 2022; Autism Spectrum Disorders Working Group of The Psychiatric Genomics Consortium, 2017). OCD PRS shows overlap in risk for anxiety, anorexia, and Tourette syndrome (Schmilovich et al., 2023). A quantitative approach across domains of ASD has clarified that Social-communicative traits (i.e., social interaction or joint attention) correlate highly, have relatively high heritability, and are intercorrelated, whereas restricted behaviors are distinct features with perhaps differential genetic architectures. These data support a partially overlapping, dimension-specific genetic risk model.

### 2.3 Spectrum heterogeneity and historical subtypes within ASD

Instead of using Asperger syndrome as an Independent Diagnostical Term, throughout this review, Asperger syndrome will be treated as a Historical Term and as a Subgroup of Autism (i.e., ASD). This treatment is consistent with Current Diagnostic Classifications, including the DSM-5 and ICD-11 Directives.

A good example of the heterogeneity within the category of ASD is Asperger syndrome; until very recently, historically characterized by preserved language and cognitive development. Genomic studies of individuals with Asperger-like profiles have identified shared risk loci for broader ASD (e.g., 3p14, 7q32) (Autism Spectrum Disorders Working Group of The Psychiatric Genomics Consortium, 2017; Schmilovich et al., 2023; Ylisaukko-oja et al., 2006) and potentially unique signals (e.g., 15q22, NTRK3) (Ylisaukko-oja et al., 2006; Salyakina et al., 2010; Warrier et al., 2015; Rehnstrom et al., 2006; Castellani and Arking, 2020), though sample sizes are often too small for definitive conclusions. GWAS studies have suggested that social-communicative (hallmarks of Asperger syndrome) traits share polygenic risk, but restricted behaviors may have their own independent variants (Satterstrom et al., 2020).

### 2.4 Rare variants and disorder-specific genetic liability

Despite these converging observations, rare variants likely remain an important source of disorder-specific liability. In ASD, *de novo* protein-truncating mutations in highly constrained genes (for example, CHD8 and SCN2A) contribute disproportionately to severe forms of the disorder (Mountford et al., 2024; Gialluisi et al., 2019). Rare variants appear to be a less considerable contribution to ADHD and dyslexia (Chen et al., 2024; Stewart et al., 2013; Piwecka et al., 2023), though the role in OCD is still emerging (Bourgeron, 2009).

Together, these results illustrate a tapestry of neurodiverse conditions intertwined at the genetic level. Common genetic variants collectively drive a large portion of liability by contributing to correlations across traits, while rare variants and polygenic substructure contribute to specificity for the condition itself. Further disentangling these layers, with both shared and discrete architecture, can lead to significant advances in understanding the biological bases for neurodiversity.

The findings of this study have been used to create a framework for continued discussion of the cellular and molecular pathways illustrated in the figure. The rationale for selecting particular genes and pathways will be further discussed by highlighting the shared geographic location of all three conditions.

## 3 Molecular and cellular pathways underpinning neurodiversity

Extending beyond genetic convergence, this section describes convergent molecular mechanisms across autism spectrum disorder (ASD), attention-deficit/hyperactivity disorder (ADHD), dyslexia, and obsessive-compulsive disorder (OCD). Integrated neuroscience is revealing convergent neurobiological structures underlying behavioral variation among these neurodiverse conditions. Although these disorders exhibit distinct behavioral phenotypes, we increasingly understand how genetic variations manifest behaviorally through disruptions in neurobiological structures, including synaptic pathways, neurodevelopment, immune-glial signaling, and aspects of chromatin regulation.

The criteria for selecting genes and pathways discussed in this section include the strength of genetic association evidence, functional relevance to the disorders, and the presence of common genetic loci across conditions. Advances in high-throughput molecular characterization (multi-omics) and functional neuroscience approaches (e.g., scRNA-seq, snRNA-seq, *in vivo*, and region-specific bulk transcriptomics) provide evidence of both shared and distinct roles of gene expression across these conditions, enhancing understanding of neurodiversity’s etiology (Jin et al., 2020; Piantadosi et al., 2021; Sudre et al., 2023).

Results show that polygenic risk, synaptic pathway disruptions, neuroimmune activation, and abnormal developmental timing are prevalent convergences across neurodiversity. Asperger syndrome, in contrast to ASD, ADHD, and dyslexia, exhibits unique gene expression patterns. Shared observations of neuroimmune pathway disruptions and glial activation suggest a neuroimmunity component influencing cognitive and synaptic plasticity; however, controversies regarding immune activation in ASD and ADHD highlight the complexities of these systems, noting both involvement and limitations.

Neurodevelopmental dysregulation, characterized by changes in synaptic pruning and altered neuronal network dynamics, is consistently reported across these disorders and contributes to understanding atypical brain circuit maturation. These molecular changes not only imply genetic convergence but also reinforce findings related to dysregulated synaptic, chromatin, and immune-regulatory genes associated with disrupted network connectivity. The identification of common loci shared between conditions reinforces the link between genetic and neurobiological findings.

Overall, these pathways demonstrate a shared neurobiological architecture that may underlie overlapping behavioral phenotypes, providing mechanistic evidence in support of the integrated framework presented.

### 3.1 Synaptic pathways and circuit-specific disruption

Synaptic pathways emerge as a unifying molecular hallmark across these disorders. In ASD, snRNA-seq of postmortem brain samples has identified significant downregulation of genes involved in synaptic vesicle cycling and postsynaptic density maintenance—especially in layer 2/3 projection neurons and VIP interneurons—while immune-related genes are upregulated in astrocytes and microglia. These transcriptomic alterations align closely with known ASD genetic risk loci, including **SHANK3**, **NLGN3/4**, and **NRXN1** (Piantadosi et al., 2021). Their mutations alter synaptic function and lead to intellectual disability, typical autism, and Asperger syndrome. *In vivo* Perturb-seq studies further confirmed that CRISPR-induced loss-of-function in high-confidence ASD risk genes disrupts transcriptional modules across excitatory neurons, inhibitory neurons, astrocytes, and oligodendrocytes, with these modules showing conservation in human cerebral organoids and ASD patient brains (Sudre et al., 2023).

OCD shares this synaptic theme, with transcriptome profiling of the striatum (caudate and putamen) revealing enrichment in synapse-associated genes, particularly those modulating glutamatergic transmission and axon guidance (Martins-Costa et al., 2024). Similarly, in ADHD, bulk RNA-seq of cortico-striatal regions highlights altered expression of genes implicated in dopaminergic signaling (e.g., **SLC6A3**, **DRD4**), synaptic scaffolding, and transcriptional regulation, mapping onto known GWAS signals (Villa et al., 2022). These differences converge on synaptic pathways but are biased toward dopamine circuitry in ADHD versus corticosteroid glutamatergic circuits in OCD.

Mutations in **CHD8**, **ARID1B**, and **SETD1A**—genes that regulate access to DNA during neurogenesis—are common to ASD (including Asperger syndrome), ADHD, OCD, and dyslexia (Gabriele et al., 2018; Naveed et al., 2024). Single-cell ATAC-seq of fetal cortex shows these genes influence cell-type-specific chromatin landscapes in proliferative and post-mitotic zones. In ASD, **CHD8** mutations are associated with altered timing of neuronal differentiation and circuit formation. OCD and ADHD demonstrate epigenetic modulation of dopaminergic and glutamatergic genes, respectively. Chromatin remodeling serves as a scaffold for condition-specific transcriptional responses to neurodevelopmental cues (Guidi et al., 2018).

Asperger syndrome, although categorized within the ASD spectrum, demonstrates both convergent and divergent molecular signatures. Transcriptomic analyses reveal distinct profiles despite shared core signaling pathways (Monet and Quan, 2023). Co-expression network analysis suggests a shift in affected pathways in Asperger syndrome, with relative enrichment for genes involved in cytoskeletal remodeling and synaptic plasticity, rather than the canonical ASD synaptic gene set. A comparative summary of key genetic loci, affected cell types, and associated molecular pathways across ASD, ADHD, dyslexia, and OCD is provided in Table 1.

**Table 1:** Comparative genetic and molecular pathways across neurodiverse conditions.

Disorder	Key genes/loci	Primary cell types affected	Core biological pathways	Functional implications
ASD	SHANK3, NLGN3/4, NRXN1, CHD8, SCN2A, DYRK1A	Layer 2/3 projection neurons, VIP interneurons, astrocytes, microglia	Synaptic vesicle cycling, postsynaptic density maintenance, chromatin remodeling, PI3K–AKT–mTOR	Impaired synaptic transmission, altered excitation–inhibition balance, delayed circuit maturation
ADHD	SLC6A3, DRD4, SETD1A, ARID1B	Cortico-striatal projection neurons, dopaminergic neurons, glia	Dopaminergic signaling, transcriptional regulation, chromatin accessibility	Dysregulated attention and impulse control, prolonged circuit maturation
Dyslexia	KIAA0319, DCDC2, ROBO1	Cortical projection neurons (perisylvian regions)	Neuronal migration, axon guidance, Wnt signaling	Disrupted phonological processing and reading networks
OCD	SLITRK5, BTBD3, SETD1A	Cortico-striatal neurons, astrocytes	Glutamatergic synaptic signaling, axon guidance, immune-glial activation	Hyperactive cortico-striatal loops, impaired cognitive flexibility
Shared across disorders	CHD8, ARID1B, SETD1A	Neural progenitors, excitatory and inhibitory neurons	Chromatin remodeling, neurodevelopmental timing control	Condition-specific transcriptional dysregulation driven by shared epigenetic scaffolds

### 3.2 Neuroimmune and glial mechanisms

A second axis of convergence involves the innate immune system and glial reactivity (Voineagu et al., 2011; Zhao et al., 2023). In ASD, single-cell transcriptomics have identified robust upregulation of immune genes in astrocytes and microglia—including complement components (e.g., **C1q**, **C3**), interferon response elements, and interleukin signaling—suggesting active neuroimmune modulation during development. Perturb-seq studies confirmed glial sensitivity to perturbations of ASD-risk genes, implicating dysregulation of astrocytes and oligodendrocytes.

Recent transcriptome-wide mega-analyses further support these findings by showing that ASD-associated transcriptional dysregulation spans both central (brain) and peripheral (blood) compartments (Sorokina et al., 2018). Co-expression network analysis revealed shared immune gene modules enriched in interferon signaling, antigen presentation, and cytokine response. These modules were co-regulated with transcription factors such as **STAT1**, **IRF1**, and **RUNX1**, suggesting a unified immunotranscriptional program.

**Figure 1:**
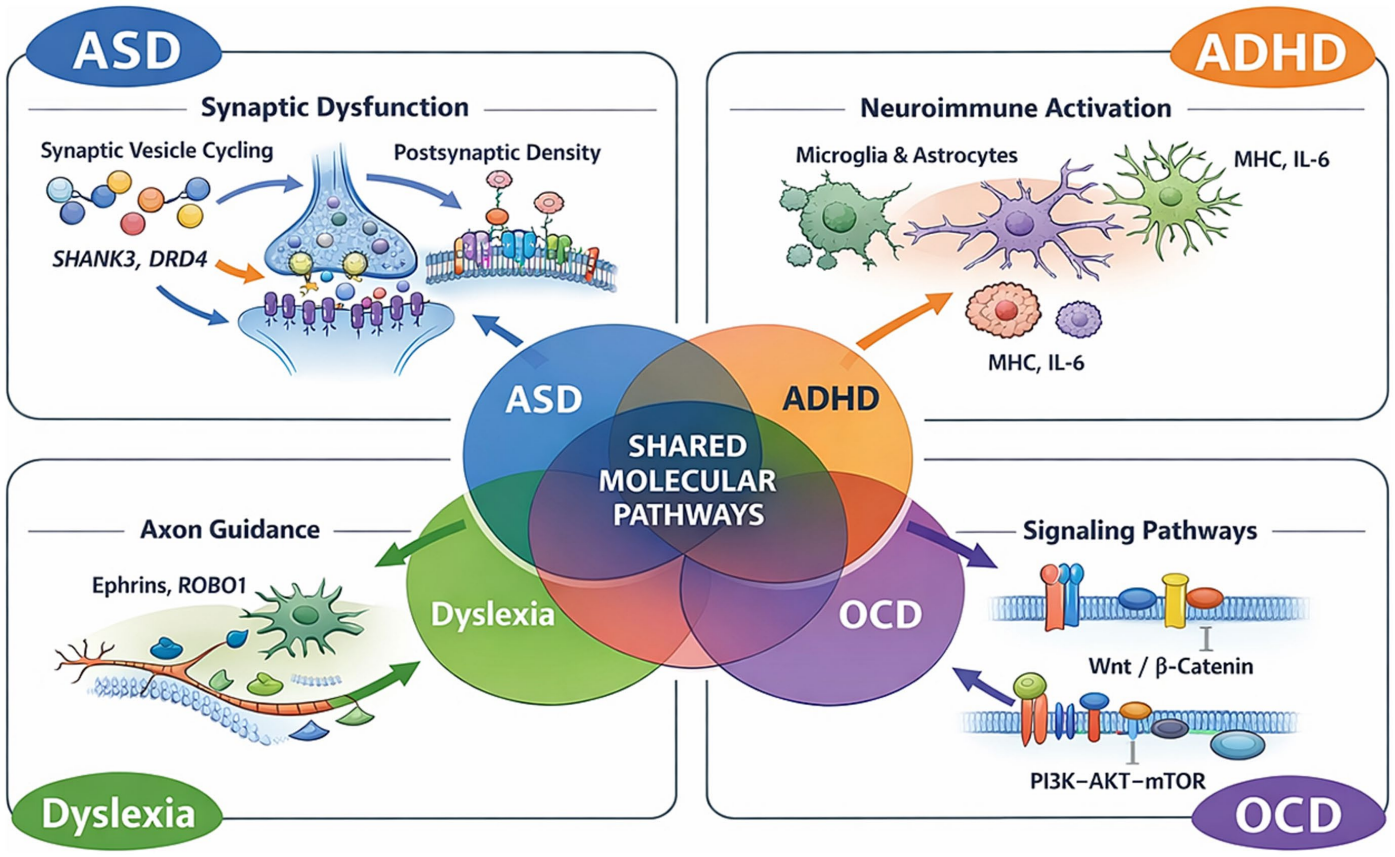
Shared and disorder-specific biological pathways across neurodiverse conditions. Schematic illustration summarizing convergent and divergent molecular mechanisms implicated in autism spectrum disorder (ASD), attention-deficit/hyperactivity disorder (ADHD), dyslexia, and obsessive-compulsive disorder (OCD). Central overlap highlights shared synaptic, neuroimmune, axon guidance, and intracellular signaling pathways, including PI3K–AKT–mTOR and Wnt/β-catenin signaling, while peripheral elements depict disorder-specific circuit and pathway perturbations.

Similarly, OCD brain samples show upregulation of **MHC-related genes** and glial markers (Kalkman, 2012), while ADHD striatal tissue reveals altered expression of cytokine-responsive transcripts (Kwan et al., 2016). These findings support a model in which neuroinflammation and immune-brain signaling reshape synaptic pruning and circuit balance in early development. In ADHD, glial expression of immune mediators may indirectly modulate dopaminergic tone, while in OCD, astrocytic reactivity may contribute to corticosteroid loop hyperactivity Figure 1.

### 3.3 Developmental signaling and transcriptional regulation

A third shared molecular domain lies in the disruption of conserved signaling cascades critical for brain development. Dysregulation of **Wnt** signaling has been observed in ASD and dyslexia, with transcriptomic studies indicating altered expression of **β-catenin** and downstream effectors in cortical progenitor and early excitatory neuron populations (Sharma and Mehan, 2021; Chen et al., 2024). The **PI3K-AKT–mTOR** pathway, frequently perturbed in ASD—including syndromic cases with **PTEN** mutations—has been implicated in OCD and ADHD (Lee et al., 2019; Velmeshev et al., 2019). Disruption of mTOR-dependent translational control may alter experience-dependent plasticity and glial function, affecting circuit maturation and cognitive flexibility.

Additionally, **axon guidance signaling molecules** such as **semaphorins**, **ephrins**, and **netrins** are implicated in long-range connectivity, particularly in dyslexia and OCD (Stiles and Jernigan, 2010). Other pathways, including **BMP/TGF-β** and **Notch**, have emerged as contributors to progenitor zone patterning, with selective disruption noted in ASD and possibly ADHD (Sicard et al., 2021).

The development of the human brain is a lengthy, multi-phase journey that occurs across the prenatal, perinatal, and postnatal periods (Horowitz-Kraus et al., 2017; Marzola et al., 2023). This journey consists of synchronized waves of neurogenesis, neuronal migration, synaptogenesis, pruning, myelination, and circuit consolidation, contributing to the emergence of sensory, motor, emotional, and cognitive domains. While neurodiverse conditions appear early in life, many continue to evolve over time. These developmental taxonomies connect genetic and molecular data to the proposed integrative framework and describe how timing and circuit development impact neurodiverse phenotypes.

### 3.4 Spatiotemporal gene expression during neurodevelopment

Neurodiverse conditions such as autism spectrum disorder (ASD), attention-deficit/hyperactivity disorder (ADHD), dyslexia, and obsessive-compulsive disorder (OCD) arise through extended developmental timelines that reflect disruptions in both the spatial and temporal dynamics of brain maturation (Miller et al., 2014; Wang et al., 2018). Large-scale spatiotemporal transcriptomic resources, including BrainSpan (Yang and Shcheglovitov, 2020) and PsychENCODE (Li et al., 2023), have demonstrated that genes implicated in these disorders exhibit tightly regulated expression patterns across specific developmental stages and brain regions, underscoring the importance of developmental timing in shaping neurodevelopmental outcomes.

In ASD, many high-confidence risk genes—including *CHD8*, SCN2A, and DYRK1A—show peak expression during mid-fetal development in prefrontal and temporal association cortices, implicating early disruptions in cortical patterning, neuronal differentiation, and synaptogenesis (Turic et al., 2010; Selten et al., 2018). In contrast, ADHD-associated genes such as *DRD4* and *SLC6A3* exhibit prolonged postnatal expression, particularly in striatal and frontal regions, consistent with the extended maturation of attention, executive control, and impulse regulation networks (Scerri et al., 2011). Dyslexia-associated genes, including *KIAA0319* and *DCDC2*, which are involved in neuronal migration and cortical layering, are preferentially expressed early in development in perisylvian and temporoparietal language-related cortices (Dhiman et al., 2025; Padmanabhan et al., 2017). OCD risk genes such as *SLITRK5* and *BTBD3* tend to peak later, aligning with the maturation of cortico-striatal-thalamic circuits that underlie cognitive flexibility and compulsive behaviors (Arnsten, 2009).

Together, these findings illustrate that neurodevelopmental risk is not solely determined by gene identity, but critically by when and where genes are expressed during brain development. Differences in spatiotemporal gene expression patterns provide a mechanistic explanation for both shared vulnerabilities and disorder-specific phenotypes across neurodiverse conditions. These coordinated temporal, molecular, and circuit-level dynamics are conceptually integrated within the multilevel framework illustrated in Figure 1, which links genetic risk to molecular pathways, developmental timing, and neural circuit maturation across neurodevelopmental disorders.

### 3.5 Circuit maturation and behavioral phenotypes

Molecular signatures—such as gene expression peaks and mutation-induced delays or accelerations in neuronal differentiation—mirror circuit-level differences. In ASD, the maturation of default mode and salience networks is delayed, correlating with the onset of social and sensory symptoms by age 2–5. In ADHD, delayed myelination and reduced connectivity in frontoparietal circuits underlie inattention and impulsivity, while dyslexia involves inefficient formation of the phonological loop—particularly among the inferior frontal gyrus and auditory cortex (Norton et al., 2015; Milad and Rauch, 2012; Miller et al., 2020). OCD follows a later trajectory, with hyperactivation of cortico-striatal loops governing cognitive flexibility and habit formation, intensifying in late childhood and adolescence (Petrelli et al., 2016).

Despite their diversity, these disorders exhibit both shared and distinct developmental trajectories (Vakilzadeh et al., 2024). Cross-disorder patterns, such as aberrant glial activation, emerge as a common feature in ASD and OCD, linking neuroinflammation with synaptic pruning during sensitive developmental windows (Geschwind and State, 2015). Increased microglial activity in the prefrontal cortex has been linked to excessive synapse elimination in ASD, while astrocyte dysregulation in cortico-striatal circuits may contribute to compulsive behaviors in OCD.

## 4 A multilevel clinical framework for neurodiversity

Genetic, molecular biological, developmental, and neural circuitry evidence suggests a multi-level integrated framework for understanding neurodivergent conditions, including Autism Spectrum Disorder (Autism) (ASD), Attention Deficit Hyperactivity Disorder (ADHD), Dyslexia, and Obsessive Compulsive Disorder (OCD). ASD, ADHD, Dyslexia, and OCD are viewed as not discrete biological entities; rather, genetically these disorders have extensive overlap in genetics, indicating they share significant polygenic risks across a continuum of transdiagnostic risk that are determined by genetic and environmental factors (Sullivan and Geschwind, 2019; Liao et al., 2025; Willsey and State, 2015; Costa-Mattioli and Monteggia, 2013). With the growing number of high-density genomic studies and Genome Wide Association Study (GWAS) findings, a growing body of literature demonstrates pervasive polygenic risk across diagnostic boundaries. The conceptual organization of this multilevel framework—linking genetic risk to molecular pathways, developmental timing, and neural circuitry—is illustrated schematically in Figure 1.

### 4.1 Multilevel integration of molecular pathways and developmental timing

There are shared convergent biological pathways that form the basis of neurodevelopmental disorders, particularly those pathways that regulate synaptic function, neuronal connectivity, cellular plasticity, and excitatory/inhibitory balance are likely pathways of the same origin that demonstrate dysregulated neurotransmitter signaling and chromatin remodeling through neuroimmune processes and intracellular signaling via multiple pathways (PI3K/mTOR, Wnt/β-catenin, MAPK) creating a mechanistic bridge between genetic vulnerability and disrupted circuit function (Duman and Voleti, 2012; Peixoto and Nelson, 2017; Cotney et al., 2015; Fletcher-Watson et al., 2021). All pathways share commonalities, but differences in timing, the extent of pathway alteration, and the cellular environment associated with altered pathway function account for the variability and heterogeneity observed across disorders such as ASD, ADHD, and other neurodevelopmental disorders. Thus, identifying genetic and molecular risk markers (e.g., CHD8, GRIN2B, FOXP2) may aid in determining and tailoring treatment strategies and developing individualized or personalized developmental plans for the recipient of treatment (Kapp et al., 2013; Doyle, 2020).

Developmental scheduling is an important aspect of this multi-level framework. The development of neurogenic cells, including the formation of synapses and the completion of circuit development, occurs within specific time frames and is highly sensitive to both genetic and environmental insults. Disruptions that occur at the initial neurodevelopmental stages of ASD have been associated with abnormal alterations in the pruning of synapses. However, later developing areas of cognitive functioning are impacted, particularly in the case of ADHD rather than ASD. Longitudinally, neuroimaging and transcriptomic studies have demonstrated that risk genes exhibit distinct spatial and temporal patterns of expression across brain regions during development, underscoring the need to align treatment strategies with the timing of the neurodevelopmental process, as well as with the individual patient’s chronological age.

### 4.2 Translational implications and clinical relevance

Genetic, molecular, and developmental processes ultimately converge to mature neural circuits, characterized by altered synaptic integrity and myelination of long-range circuit connections, resulting in atypically organized neural circuits that generate patterns of behavior. Although the clinical presentations of ASD, ADHD, and other neurodevelopmental disorders are distinct from one another, they have a number of common vulnerabilities that explain many of the overlapping cognitive/behavioral features found across neurodiverse populations. Translationally, interventions that aim to improve the adaptive neuroplastic abilities of an entire circuit system, rather than focusing on an isolated genetic or individual pathway, are more likely to result in substantial functional improvement across a larger number of neurodiverse individuals, consistent with the principles emerging from the practice of Precision Medicine (Kapp et al., 2013; Doyle, 2020).

## 5 Conclusions and future perspectives

In conclusion, this integrated multi-level perspective on neurodiversity allows us to view it as a biologically interconnected continuum of neurodevelopmental diversity, NOT as defined by separate diagnostic entities. This model of neurodevelopmental diversity helps to integrate the continuum of genetic vulnerability, biological mechanisms associated with the development of the brain through the timing of development to completion of neural circuit maturation, to assist us in better understanding the heterogeneity associated with recognizing the relationship between genetic predisposition and molecular biological mechanisms across all neurodevelopmental conditions. Future research efforts should focus on longitudinal, multidisciplinary studies integrating Genomics, Neurodevelopment, and Behavioral Neuroscience to support the validation and refinement of this multi-level framework and how to establish evidence-based methods of care, an individualized approach to care, and promote equitable access to care throughout the individual life span (Doyle, 2020).
